# Supplementary material for: Are carbon emissions trading and green financial instruments synergistic? -Comprehensive quantitative research based on content analysis
Source: PLoS One. 2024 Mar 7;19(3):e0298601. doi: 10.1371/journal.pone.0298601 (PMC10919601; doi:10.1371/journal.pone.0298601)
Supplement: S1 Data — (ZIP) [file pone.0298601.s001.zip › Data/地级市Z_F工作报告.zip!/║■▒▒/╗╞╕╘╩╨/└┤╫╘╣1⁄2╓┌║┼í╛┬φ┐╦╩2╛▌═°í┐_2009.docx]

各位代表：

　　现在，我代表市人民政府向大会报告工作，请予审议，并请市政协各位委员和其他列席人员提出意见。

　　一、2008年工作回顾　　2008年是极不平凡的一年。面对历史罕见的雨雪冰冻灾害和严峻复杂的国际国内经济形势，在市委正确领导下，市人民政府团结带领全市人民，深入贯彻落实科学发展观，抢抓机遇，加快发展，全市经济呈现增长加快、结构趋优、动力增强、民生改善的良好格局，各项社会事业全面进步。除居民消费价格指数外，本届人大三次会议确定的主要预期目标全面超额完成。

　　市域经济全面增长。全市地区生产总值600.8亿元，比上年增长15%。财政收入59.6亿元，增长31.7%。其中地方一般预算收入26.2亿元，增长31.5%。全口径财政收入、地方一般预算收入、全社会固定资产投资、规模以上工业增加值、外贸出口实现三年翻番。固定资产投资、社会消费品零售总额、工业增加值、地方一般预算收入的增幅位居全省前列。主要经济指标首次实现了赶超全省平均发展水平的目标。农业增产增效。主要农产品全面增产，优质稻、桑茶药、油菜、花生、生猪、蛋鸡、小龙虾、速生丰产林等特色板块和畜牧水产大县建设加速推进。新认定国家地理标志保护产品4个、无公害农产品27个。工业加速成长。规模以上工业企业达到1003家，当年新增208家，提前两年实现“十一五”目标;规模以上工业增加值141.3亿元，增长30.6%。当年获得中国驰名商标2个，湖北名牌产品10个。现代服务业加快发展。社会消费品零售总额282.3亿元，增长26.2%。金融机构各项存款余额635.1亿元，增加134.4亿元;各项贷款余额252.4亿元，增加34.3亿元。房地产投资27.4亿元，增长17.3%。接待游客585万人次，创旅游收入25.5亿元，增长15.8%和15.9%。

　　发展方式加快转变。三次产业协同带动。第一产业增加值192.6亿元，增长7.1%;第二产业增加值204. 2亿元，增长20%;第三产业增加值203.9亿元，增长17.4%。投资拉动作用明显增强。全社会固定资产投资370.9亿元，增长43.5%。其中城镇以上投资315.8亿元，增长51.4%。城镇以上新开工项目1335个，比上年增加664个。交通建设，完成通村油路2583公里，武英、武麻、大广北高速公路、鄂东长江大桥建设快速推进。武汉新港唐家渡作业区建设正式启动。武合快速铁路投入试运营，京九铁路和汉麻铁路电气化改造工程全线开工。能源建设，大别山电厂一期工程顺利投产，白莲河抽水蓄能电站即将并网发电，供变电能力进一步提升。农田水利建设，长江回水堤防整险加固工程基本完工，病险水库除险加固工程完成20座，解决了55.6万人饮水安全问题，高产农田建设和低丘岗地改造工程、水库移民后扶工程等有序进行。城乡建设，市区以遗爱湖治理保护工程和道路改造刷黑工程为重点，深入推进“四城联创”;县市城区、小城镇和新农村建设以争创“楚天杯”和“十镇百村”示范工程为载体，规划、建设与管理全面加强。科技对经济的贡献率进一步提高。加强市校合作，实施各类科技计划项目60项，新增技术改造资金1.3亿元。全市高新技术产业实现增加值29.8亿元，增长31.3%。节能减排和安全生产深入推进。市区和武穴污水处理厂建成投产，“九小”企业关停任务提前超额完成，重点耗能和排污大户整治措施基本落实。土地、水、森林、矿产等资源保护力度加大。全市单位生产总值能耗同比下降4.6%，化学需氧量和二氧化硫排放量净削减1.72%和1.84%。落实安全生产责任制，加强隐患排查整治，安全生产各项指数都控制在省政府下达的责任目标范围内。产品质量和食品药品安全监管等工作取得明显成效。

　　改革开放不断深化。扎实推进“两型”社会综合配套改革，制定了总体方案和配套规划体系，争取了一批重点启动项目。在国家扩大内需首批1000亿元投资中，我市已争取项目资金5.2亿元，居全省市州首位。农村公益性服务“以钱养事”新机制不断完善，集体林权制度改革、国有农场管理体制改革以及粮食流通、农资购销等体制改革全面展开，水管体制改革如期完成。政策性“三农”保险试点、集体资产产权制度创新试点、农村土地管理制度创新、现代农村金融制度创新等有序进行。邮政储蓄银行组建完成。金融对经济的支持作用进一步增强。农民专业合作组织蓬勃兴起。城镇居民基本医疗保险和黄冈城区被征地农民基本养老保险改革全面启动。部门预算制度渐趋规范。取消和调整了一批审批项目，行政服务中心效能进一步提高。土地收购储备和“招拍挂”出让制度全面建立，城投公司和金融合作机构的职能作用有效发挥。商贸企业和部门企业改革扎实推进，国有资产管理进一步加强。大力推进全民创业，当年新增个体经营户2.73万户、私营企业1464家，新增注册资金20.3亿元。对外开放取得积极成果。当年引进1000万元以上项目679个，亿元以上项目103个，过10亿元项目7个，新开工外来投资项目453个，完成投资54.4亿元。实际利用外资1.34亿美元，增长13.6%。外贸出口4.95亿美元，增长36.7%。各类开发区和工业园区在改革开放、招商引资等方面发挥了示范效应。

　　民生问题逐步改善。实施积极的就业政策，当年新增城镇就业6.2万人，下岗失业人员再就业2.5万人，帮助“4050”人员再就业1.3万人，组织农村劳动力转移就业18万人。全市城镇登记失业率为4.07%，低于年度控制目标0.43个百分点。社会保险参保总人数144.5万人，征收保险费12.34亿元。城镇居民基本医疗保险全面启动，参保人数达82.9万人，参保率达57.1%。11189户因灾倒房全部恢复重建。为14.2万城市低保对象发放低保金2.2亿元，为22.3万农村低保对象发放低保金1.2亿元，城乡低保人均月补差分别提高44元和14元。加强价格管理，市场物价逐月回落，年末全市居民消费价格指数涨幅为6.42%。加强流浪乞讨人员救助管理，扩大医疗救助受惠面，多途径组织残疾人就业，加快农村福利院升级改造。加强经济适用房和廉租住房建设，开工建设经济适用房26.5万平方米，筹集廉租住房5.4万平方米。加大扶贫开发力度，解决5万农村特困人口的温饱问题，帮助8万低收入贫困人口摆脱贫困。积极募集款物并参与四川地震救灾，对口支援汉源县重建项目进展顺利。切实加强农民负担管理，维护劳动者的合法权益，城乡居民收入再创历史新高。全市城镇居民人均可支配收入9952元，人均增加1637元，增长19.7%;农民人均纯收入3744元，人均增加449元，增长13.6%。

　　社会事业协调发展。完善义务教育经费保障机制，落实义务教育阶段“两免一补”资金4.7亿元。落实中职生生活补助资金7343万元，办理大学生生源地信用助学贷款5387万元。素质教育扎实推进，基础教育、职业教育、高等教育均衡发展。加强公共卫生体系建设，新型农村合作医疗实现全覆盖，平均参合率达到90.9%，城镇社区卫生服务得到加强。积极开展“问题奶粉”患儿救治工作，手足口病、艾滋病、血吸虫病、结核病防控能力提高。落实“两控五规范”措施，加强人口与计划生育工作。全市人口出生率为8.06‰,符合政策生育率达到95.09%。继续实施广播电视、电话“村村通工程”和“农村信息化示范工程”，积极推进数字电视整体转换。首次投资拍摄的电影《黎明行动》在全国公开上映。实施文化惠民工程，建成标准农家书屋628个，新建和扩建乡镇文化站33个。以“迎奥运”为主题的群众性体育活动和送体育下乡活动蓬勃开展。民族、宗教、外事、侨务、对台、人防、保密、档案、气象、地震、方志、经研、妇女、儿童、老龄等各项工作都取得了新的成绩。

　　民主法制和政府执行力建设得到加强。认真落实国务院、省政府关于加强市县政府依法行政的决定，大力提高行政机关工作人员依法行政的意识和能力，完善政府行政决策机制，建立健全规范性文件监督管理制度，深入开展基层执法单位文明执法教育活动，法治政府建设迈出新步伐。加强社会治安综合治理和“平安黄冈”建设，依法严厉打击各类犯罪，保持了社会稳定。深入开展群众性精神文明创建活动和文明城市创建活动，公民思想道德素质和城乡文明程度进一步提高。大力开展国防动员和双拥工作。加强应急管理和群众来信来访工作，加强政府信息公开和电子政务建设，加强行政监察和审计监督，加强源头治腐和勤政廉政建设，深入开展政府执行力大讨论，倡导“八简”新风，学习企业文化，完善工作机制，政府效能不断提升，机关作风有了明显变化。

　　各位代表!在本届人大三次会议上，市政府承诺办好涉及城乡居民就业、就学、就医、出行、住房等8个方面的实事，经过一年来的努力，均已圆满完成或超额完成。其中，就业再就业和劳动力转移培训、农村公路和客运市场体系建设、农村合格学校建设、困难群众法律援助、城市社区卫生服务体系建设、经济适用房和廉租住房建设等项目超额完成;《黄冈城区被征地农民基本养老保险试行办法》开始实施;灵活就业人员养老保险补贴和基本医疗保险优惠政策得到落实;市区重点建设工程按既定计划推进。

　　过去的一年，我市经济社会发展取得的成绩来之不易。这是党中央、国务院、省委、省政府和市委正确领导、科学决策的结果，是全市人民不畏艰难、团结奋斗的结果，是市人大、市政协监督支持，各民主党派、工商联、无党派人士，各人民团体，中国人民解放军驻黄部队、武警部队、海内外黄冈同乡、各界朋友和投资客商鼎力相助的结果。在此，我代表市人民政府，向全市广大干部群众、向所有关心支持黄冈建设发展的海内外人士，表示衷心的感谢!

　　在充分肯定成绩的同时，我们也清醒地认识到面临的困难和问题。主要是：国际金融危机对实体经济的影响不断加深，部分行业企业生产经营困难，利用外资和出口难度加大;经济总量偏小与产业层次不高的问题并存，面临着加快发展与转变发展方式、增加就业岗位等多重压力;农产品价格下行压力较大，农民务工出现新的困难，农民持续增收困难增大;财政政策性减收因素增多、刚性支出增大，社会事业发展欠账较多，一些关系群众切身利益的问题需要继续下大力解决;政府职能需要加快转变，行政效能有待提高。对这些问题，我们将进一步增强忧患意识和责任意识，采取积极有效的措施，努力加以解决。

　　二、2009年政府工作的指导思想和奋斗目标　　2009年是新中国成立六十周年，是推动经济平稳较快增长和社会和谐稳定的关键年。今年政府工作的指导思想是：认真贯彻党的十七大和十七届三中全会精神，深入贯彻落实科学发展观，抢抓国家实施扩大内需政策措施、武汉城市圈“两型社会”综合配套改革试验和沿海产业转移等重大机遇，以保增长为首要任务，以改善民生为根本目的，进一步解放思想、坚定信心，加大项目建设和招商引资力度，加快转变发展方式和调整经济结构，加速推进体制机制改革和扩大对内对外开放，加强城乡建设和社会建设，努力实现经济社会又好又快发展。

　　主要预期目标是：地区生产总值增长12%以上，规模以上工业增加值增长20%以上，全社会固定资产投资增长25%以上，社会消费品零售总额增长18%以上，吸收外商直接投资增长15%，外贸出口增长15%，地方一般预算收入增长15%;单位生产总值能耗下降4.8%，化学需氧量和二氧化硫排放量分别下降2.5%和0.8%;城镇居民人均可支配收入增长10%，农民人均纯收入增长8%;城镇登记失业率控制在4.5%以内，居民消费价格涨幅控制在4%左右，人口自然增长率控制在6‰以内。

　　上述经济社会发展目标，是辩证分析当前经济形势、综合考虑各方面因素提出来的。当前，我们面对的挑战前所未有，机遇也前所未有。国际金融危机仍在扩散和蔓延，对我市经济发展的影响将更加明显，我们面临的形势比以往更加复杂，更加严峻。我们一定要把困难估计得更充分一些，把应对措施考虑得更周全一些，把各项工作做得更扎实一些，防止由于估计不足和准备不够而陷入被动。同时，我们更要看到，黄冈可持续发展具备许多有利条件和积极因素。面对挑战，应对危机，措施千万条，信心最重要。我们的信心来自于党中央、国务院出台的扩大内需、促进经济增长的一系列政策措施，来自于党的十七届三中全会推进农村改革发展的重大部署，来自于省委、省政府为推进武汉城市圈“两型社会”综合配套改革而谋划、实施的工作方略和重点项目，来自于国外和沿海产业加速向内地转移的重要契机，来自于黄冈改革开放30年特别是近几年夯实的发展基础，来自于通过激励竞争形成的县域经济竞相发展的良好态势，更来自于黄冈老区干部群众思进取、谋发展、图赶超的决心和干劲。只要我们坚定信心、埋头苦干，审时度势、趋利避害，就能够战胜一切艰难险阻，牢牢把握科学发展的主动权，努力开创黄冈改革发展的新局面。

　　三、努力推动经济平稳较快发展　　坚持保增长、扩内需、调结构、促就业、重民生、强基础，抢抓发展机遇，拓展发展空间，破解发展难题，增强发展后劲，奋力实现更长时间、更高水平、更好质量地发展。

　　(一)千方百计扩大投资促进消费

　　狠抓项目建设，扩大投资规模。深入开展“大干项目年”活动，抓紧策划、争取和实施一批影响全局、支撑长远发展的重大项目。重点抓好武麻高速、武英高速、大广高速、京九铁路和汉麻铁路电气化改造、白莲河抽水蓄能电站、雨润食品、李时珍国际健康旅游、祥云化工等重大续建项目，抓紧启动武汉至黄冈城际铁路及黄冈长江(公铁)大桥、武汉新港罗霍洲作业区、麻竹高速、黄州火车站升级改造、华海造船、贝因美食品、亚东水泥、鸿路钢构、济氏药业和九江长江大桥连接内线等新开工项目，强力推进林纸一体化、浠水核电、黄冈电厂、大别山电厂二期、西隔堤加固、大别山红色旅游公路、麻阳高速等项目前期工作。与此同时，深化投资体制改革，完善重大项目推进工作机制和协调服务机制，鼓励和带动民间投资持续增长，确保项目早竣工、早投产、早见效。

　　扩大消费需求，活跃城乡市场。增强城乡居民消费能力。认真落实国家增加城乡居民收入的有关政策，提高企业离退休人员养老金标准和城乡低保保障水平，提高群众特别是困难群众的收入，扩大即期消费。改善消费环境。加强价格调控和监管，加强以食品药品为主的产品质量监管，健全产品质量安全检测体系，严厉打击损害消费者权益的行为。加大廉租房、经济适用房等保障性住房的开发建设，鼓励居民购房，促进房地产业稳定健康发展。积极发展服务业。围绕建设大别山旅游经济带，改善设施条件，加大市场促销，培育旅游精品，创建旅游名镇，提升黄冈旅游竞争力。大力实施“万村千乡市场工程”和“新农村现代流通服务网络工程”，加强邮政服务“三农”站点建设，深化“农超对接”，推进“家电下乡”。提升传统服务业，拓展现代物流业，积极发展新型服务业。

　　多方融通资金，支持经济增长。发挥财政资金的政策引导作用，做好增值税转型改革以及其他税收政策调整的组织实施工作，减轻企业税负，增强企业投资意愿和能力。做实做强信用担保平台，支持金融机构调整优化信贷结构，加快金融产品和服务创新，增加有效信贷投放。优化金融生态环境，加强银企合作，着力解决重大项目和中小企业贷款难问题。拓宽直接融资渠道，大力推进企业上市。

　　(二)突出重点发展工业经济　　加大规模产业和企业培育力度。继续实施100亿产业、100亿工业园区、100家重点企业、100个重点项目、新增100家规模以上工业企业、市县领导联系100家民营企业等“六个一百”工程，着力打造纺织服装、医药化工、食品饮料、新型建材、钢构、造船、能源等百亿产业，以重点产业的突破带动工业规模的快速扩张。通过资产重组、资源整合，培育发展一批核心竞争力较强的集团，打造代表黄冈形象的领军企业。着力实施“中小企业成长工程”，千方百计为企业排忧解难，提供政策扶持和优质服务。放宽市场准入，优化投资环境，毫不动摇地鼓励、支持、引导非公有制经济发展，深入推进全民创业。

　　加大产业集群和工业园区培育力度。重点推进武穴医化、蕲春药材、麻城汽配、团风钢构、市直食品饮料等全省重点产业集群的链条延伸，带动上下游产业发展。将产业集群建设、工业园区建设和园区招商工作有机结合起来，科学定位园区、规划园区、建设园区，推进园区产业联合、利益连接和税收共享，提高园区建设水平。把工业园区作为城镇建设的拓展点和全民创业的新空间，着力推进乡镇工业集中区建设，实现园区建设与城镇建设良性互动。

　　加大精品名牌培育力度。大力开展“质量兴市”活动，认真落实市政府出台的奖励政策，以企业为主体，发挥相关部门的支持、引导和保障作用，着力打造一批在国内外有影响、市场占有率高的工业品牌。加强企业素质样板工程建设，引导企业加强管理、苦练内功，增强抗御市场风险能力。

　　加大企业创新能力培育力度。深入开展市校合作，扶持一批经济实力雄厚、科研开发能力强的企业创办技术中心，与高等院校、科研院所开展产学研合作。以推进企业信息化为突破口，加快高新技术和先进制造技术在纺织、机械、化工等传统产业的应用，加快传统产业改造升级。充分发挥政府科技投入的引导作用，加强高新技术产业化示范基地、成果中试基地、科技企业孵化器建设。

　　(三)保障农产品有效供给和促进农民增收

　　加快发展现代农业。按照“标准化生产、连片集中、统一品种”的要求，对优势农产品板块基地进行提档升级。加快高产农田建设、低丘岗地和低效林改造、现代农业示范区建设步伐，加快以奶牛牧场为重点的畜牧养殖小区和水产片带建设。加强品种改良和疫病防控。创新农业产业化经营模式，推动金融资本、产业资本和技术要素以股权形式进入农业。在税收、用地、用电、信贷、上市等方面，支持龙头企业加快发展。

　　稳定完善农村基本经营制度。赋予农民更加充分而有保障的土地承包经营权，现有农村土地承包关系保持稳定并长久不变。建立土地承包经营权流转市场和农村集体建设用地流转市场，按照依法、自愿、有偿原则，允许农民以转包、出租、互换、转让、股份合作等形式流转土地承包经营权，发展多种方式的土地适度规模经营。大力发展农民专业合作组织。

　　加强农业基础设施建设。重点推进长河流域水生态系统修复保护工程、华阳河流域综合治理工程、中小河流治理工程、大别山南麓革命老区水土保持工程、病险水库除险加固工程、灌区节水改造与续建配套工程、泵站更新改造工程，大力实施农村饮水安全工程、移民工程、农村沼气工程和气象现代化建设。加快推进农业机械化。

　　多途径促进农民增收。认真执行国家较大幅度增加农业补贴、扩大补贴范围、较大幅度提高粮食最低收购价、解决农民工就业等一系列强农惠农政策。落实促进务工农民市民化的各项措施，推进城乡劳动就业一体化，最大限度安置好农民工,努力增加农民的务工收入。落实农民工返乡创业扶持政策,抓好“回归创业工程”和返乡农民创业服务促进工作，促进“打工潮”向“创业潮”转变。

　　继续推进扶贫开发。抓紧实施448个村的整村推进，加强产业化扶贫，以支柱产业和龙头企业带动贫困地区和贫困农户发展生产。大力扶持贫困村的村组道路、农田水利、医疗卫生、危房改造等基础设施建设，改善贫困地区群众的生产生活条件，解决5万特困人口和15万低收入贫困人口的温饱和脱贫问题。

　　(四)统筹区域经济发展

　　加快壮大市区龙头经济。坚持市区一体，理顺和创新体制机制，完善利益分配关系，加快南湖工业园、火车站化工园、禹王工业区等园区建设步伐，增强市区的集聚、辐射功能和综合承载能力。做实做强以饮品、造船、窑炉、造纸、机械电子等为重点的产业集群，以房地产业、物流业、旅游业为主体的现代服务业，以蔬菜、奶牛、水产为重点的高效农业，确保主要经济指标高于全市平均发展水平。加强城市规划、建设与管理，深入开展“四城联创”，努力实现省级卫生城市创建达标;推进城市管理综合执法，相对集中行政处罚权，依法治理违章建设;搞好城市经营，多方筹措资金，挖掘自然人文资源，提升城市品位。

　　加速发展县域经济。立足县域优势资源和产业基础，着力提升县域工业园区，千方百计抓项目、增投资，抓主体、上水平，抓集群、强实力，抓结构、促升级，着力培育一批销售收入过亿元的中小企业和销售收入过50亿元、过100亿元的县域工业园区。加强县市城区建设，努力改善城市面貌和人居环境，增强统筹城乡发展的能力。落实发展县域经济的政策措施，完善考评指标体系，健全激励竞争机制，力争有1—2个县市区进入全省县域经济20强。

　　加强小城镇和村庄建设。完善乡镇土地利用总体规划和村镇规划，抓好城镇和乡村道路、绿化、污水处理和垃圾处理等基础设施建设。坚持产业兴镇，强化项目带动，加快长江沿岸、京九沿线和大别山腹地城镇带的兴起。引导农民住宅建设按规划、有计划地逐步向中心村集中，并与村庄改造、土地整理相结合，鼓励集中建设农民新村。防治农业面源污染，大力推进“清洁家园”建设。

　　四、坚定不移地推进改革开放

　　加快“两型社会”综合配套改革试验。全面启动重点改革试验三年行动计划，围绕土地、金融、环保等方面的体制机制创新，推动改革试验具体化、项目化。围绕建设沿江经济开发带和大别山旅游经济带，重点抓好产业园区互动发展试点、产业集群发展新机制试点、循环农业和生态农业示范园区试点，抓紧黄冈临港经济先导区和市区水生态保护治理试点的规划编制与论证等前期工作。强化节能减排工作责任制，全面推行排污许可证制度，严格执行项目开工建设环评审批和节能评估审查，继续开展“九小”专项治理，坚决遏制高耗能、高排放行业过快增长。开展全民节能行动，加强冶金、建材、化工、电力等重点行业的节能改造。着力抓好绿色无公害农产品基地、湿地保护、森林保护、长江防护林、退耕还林、地质环境保护、清洁节能等生态项目，大力发展循环经济，办好一批“两型社会”示范园区、企业和村镇。

　　深化国有企业改革和事业单位改革。围绕建立现代企业制度，妥善解决改制企业的历史遗留问题，在债务处置、职工安置、完善社保等方面加大工作力度，巩固改制成果。进一步推进商贸企业和部门企业改革。加快政企、政资、政事、政府与中介组织职能分开步伐，完善国有资产监管体系，提高国有资本经营绩效。分类实施事业单位改革。

　　推进农村改革。积极创造条件，探索建立大别山新农村建设试验区，带动全市农村改革发展。深化农村配套改革，巩固和完善农村公益性服务“以钱养事”新机制。放宽农村金融准入政策，加快建立商业性金融、合作性金融、政策性金融相结合的农村金融体系。大力发展村镇银行，开展小额贷款公司试点，允许有条件的农民专业合作社开展信用合作。全面推进集体林权制度改革，基本完成集体林权确权到户任务。继续推进城中村、城郊村、园中村集体资产产权制度创新工作。

　　深化财政改革。围绕推进基本公共服务均等化，完善公共财政体系。深化预算管理体制改革，强化预算管理和监督，增强预算管理的刚性和透明度。优化财政收支结构，提高财政收入质量。坚持依法治税，强化收入征管，确保圆满完成财税任务。继续清理行政事业性收费和政府性基金，加强规范管理。完善国库集中收付运行机制。扩大政府采购的范围和规模。

　　坚定不移地实施开放先导战略。创新招商引资方式方法，大力推进产业招商、园区招商、节会招商、委托招商，用市场的办法支持企业招商，用激励的办法引导全员招商，用开放的办法推动全方位招商，广泛宣传黄冈，诚招天下客商，积极承接沿海产业转移，努力提升招商引资质效。优化出口结构，培育出口品牌，抓好一批出口基地建设，促进出口稳定增长。加快实施“走出去”战略，扩大境外工程承包、经济技术合作和劳务输出。

　　五、加强以改善民生为重点的社会建设

　　多渠道扩大就业。实施积极的就业政策，大力推进创业带动就业，开发更多的公益性就业岗位。完善公共就业服务体系，加强职业技能培训，加强就业援助，重点帮扶就业困难人群和零就业家庭。充分发挥失业保险调控和促进就业的功能，积极开展援企稳岗工作，引导企业不裁员或少裁员，保持就业局势基本稳定。

　　加快完善城乡社会保障体系。继续加大社会保险扩面征缴力度，做好城镇职工基本养老保险省级统筹相关工作，实施农业“小三场”、国有林场职工参加基本养老保险工作，开展新型农村社会养老保险试点。完善覆盖城乡的基本医疗保障体系，城镇居民医疗保险参保率达到80%，新型农村合作医疗参合率稳定在90%以上。完善住房公积金制度，全市住房公积金覆盖率达到68%。完善城乡低保制度，做好社会救助工作，保障和改善困难群众基本生活。

　　努力提高教育和医疗服务水平。完善和巩固九年制义务教育，重视学前教育和特殊教育，加大对高中阶段教育支持力度，大力发展职业教育，努力提升高等教育质量和服务社会能力。继续化解农村“普九”债务。实施人才强市战略，加强技能型和创新型人才的培养、引进和使用工作。积极推进医药卫生体制改革，加强农村医疗卫生服务体系建设，发展社区卫生服务。加强艾滋病、血吸虫病等重大传染病防治工作，提高人民群众健康水平。

　　大力发展文化事业和文化产业。加强社会主义核心价值体系建设，大力弘扬民族精神和时代精神，深入开展群众性精神文明创建活动。大力发展公益性文化事业和经营性文化产业，加强广播电视“村村通”、文化信息资源共享工程农村基层服务点、农村电影放映工程等重点文化惠民工程建设。发展社区文化和群众体育，丰富群众文化生活，推出一批文艺精品。办好湖北省第七届黄梅戏艺术节。重视支持新闻出版工作。做好文物普查工作。积极筹建市级文化体育设施。

　　切实加强社会管理。加强人口与计划生育管理，稳定低生育水平，深入开展关爱女孩行动，综合治理出生人口性别比。高度重视农产品、食品药品质量安全。推进农业标准化生产，加强农产品安全监管。开展食品药品专项整治，杜绝重大食品药品安全事故。严厉打击假冒伪劣行为，规范市场秩序。严格统计监测评价考核，做好第二次经济普查登记工作。继续做好二轮志书编修工作。认真做好民族、宗教、外事、侨务、对台、保密等工作，重视支持老龄和残疾人事业。

　　坚决维护社会和谐稳定。健全信访工作责任制，妥善处理人民内部矛盾，坚持大接访，开展大调解，及时发现和解决各类矛盾纠纷。完善应急管理体制机制，加强预警预测，妥善处理群体性事件，全力做好抗灾救灾避灾工作。落实安全生产责任制，完善规章制度，加强科学管理和严格监督，加大消防安全工作力度，彻底排查治理各类隐患，坚决遏制重特大事故发生。加强国家安全工作。加强社会治安综合治理，加大警务信息化建设力度，健全社会治安“大防控”体系，积极预防青少年犯罪，深入开展“平安黄冈”创建活动，依法打击违法犯罪行为，增强人民群众的安全感。深入开展国防教育，支持驻黄解放军和武警部队建设，不断提高预备役部队、民兵和人民防空建设水平。积极开展双拥共建活动，巩固军政军民团结。

　　努力为人民群众办实事。进一步加大投入、提高标准、落实措施，着力解决好人民群众普遍关心的实际问题：(1)城镇新增就业4万人，培训劳动力30万人次。(2)新增廉租房10万平方米。(3)新建通村水泥(沥青)公路2000公里。(4)再解决农村50万人饮水安全问题。(5)实施农村特困户和贫困残疾人家庭危房改造2000户;为1000名贫困白内障患者实施复明手术。(6)新建农村户用沼气6万户，联户和养殖小区沼气工程200处，乡村沼气服务网点460个。(7)再建成标准农家书屋400个，配套建设乡镇综合文化站19个;新建“农民体育健身工程”100个;新建或改造乡镇和村级农家店500个。(8)继续改造农村薄弱学校，建成合格学校210所;实施市区中小学危房改造改建。(9)为困难群众和农民工办理法律援助案件1000件、法律援助事项10000件以上。(10)县市城区全面建成污水处理厂及配套管网;市区争取开工建设垃圾无害化处理厂、新建4座小型垃圾中转站和10座公厕。

　　六、加强政府自身建设　　深入学习实践科学发展观，加快黄冈老区经济社会发展，必须全面加强政府自身改革与建设，努力建设人民满意的政府。

　　推进依法行政。自觉接受市人大及其常委会的法律监督、工作监督和市政协的民主监督，高度重视人大代表建议和政协委员提案的办理工作。认真听取各民主党派、工商联、无党派人士的意见，支持工会、共青团、妇联等群团组织开展工作。深入开展“法治黄冈”建设，扎实推进“五五”普法。扩大基层民主，完善政务、厂务、村务公开。加大政府信息公开力度，抓紧建成电子政务专网。健全政府重大决策的规则和程序，认真落实调查研究、专家论证、公众参与、合法性审查和集体决策制度。加强和改进行政执法、层级监督、行政复议、规范性文件管理等政府法制工作。完善行政监督机制，健全社会监督和舆论监督。

　　提高服务质量。进一步转变政府职能。减少行政审批事项，规范行政审批行为，切实加强和改善市场监管，维护社会公正和秩序，把重点放到为市场主体服务、营造发展环境上，把着力点更多地放到解决民生问题上。进一步提高政府效能。倡导“马上就办、有请必复”的办事理念和机关文化，定下来的事雷厉风行，看准了的事一抓到底。学习优秀企业的文化理念、科学管理和工作效率，节约行政成本，千方百计减费用、减时间、减程序，大力压缩各种会议、文件，精简各类检查、评比表彰，建设运转高效、办事干练、充满活力、富有成效的政府机关。进一步增强行政能力。领会和把握科学发展观的深刻内涵，学习新知识，研究新情况，解决新问题，用市场的办法、开放的办法、改革的办法破解发展难题，打破常规、超常工作，提高抓落实、办成事的能力。

　　保持清正廉洁。坚持为民、务实、清廉，弘扬黄冈老区精神，增强公仆意识和宗旨观念，带头过紧日子，严格控制一般性支出。着力推进政府系统惩治和预防腐败体系建设，健全行政服务网络，建立完善电子监察系统和综合招投标中心，加大对重点领域、重点部门、重点项目、重点资金的检查监督力度。加强领导干部经济责任审计，全面实行行政领导人员问责制度，坚决纠正有令不行、有禁不止现象，严肃查处各种腐败行为，始终保持昂扬向上、奋发有为、清正廉洁的政治本色。

　　各位代表!新的一年，我们的任务繁重而艰巨，我们的责任重大而光荣，我们的前景光明而美好。让我们更加紧密地团结在以胡锦涛同志为总书记的党中央周围，在市委正确领导下，认真贯彻落实科学发展观，坚定信心，克难奋进，全面完成今年的各项任务，以优异成绩向新中国成立六十周年献礼!
